# Choroidal layer segmentation in OCT images by a boundary enhancement network

**DOI:** 10.3389/fcell.2022.1060241

**Published:** 2022-11-10

**Authors:** Wenjun Wu, Yan Gong, Huaying Hao, Jiong Zhang, Pan Su, Qifeng Yan, Yuhui Ma, Yitian Zhao

**Affiliations:** ^1^ Ningbo Cixi Institute of Biomedical Engineering, Ningbo Institute of Materials Technology and Engineering, Chinese Academy of Sciences, Ningbo, China; ^2^ University of Chinese Academy of Sciences, Beijing, China; ^3^ The Affiliated Ningbo Eye Hospital of Wenzhou Medical University, Ningbo, China; ^4^ School of Control and Computer Engineering North China Electric Power University, Baoding, China; ^5^ Zhejiang Engineering Research Center for Biomedical Materials, Ningbo Cixi Institute of Biomedical Engineering, Ningbo Institute of Materials Technology and Engineering, Chinese Academy of Sciences, Ningbo, China

**Keywords:** choroidal layer, optical coherence tomography, boundary segmentation, deep learning, high myopia

## Abstract

Morphological changes of the choroid have been proved to be associated with the occurrence and pathological mechanism of many ophthalmic diseases. Optical Coherence Tomography (OCT) is a non-invasive technique for imaging of ocular biological tissues, that can reveal the structure of the retinal and choroidal layers in micron-scale resolution. However, unlike the retinal layer, the interface between the choroidal layer and the sclera is ambiguous in OCT, which makes it difficult for ophthalmologists to identify with certainty. In this paper, we propose a novel boundary-enhanced encoder-decoder architecture for choroid segmentation in retinal OCT images, in which a Boundary Enhancement Module (BEM) forms the backbone of each encoder-decoder layer. The BEM consists of three parallel branches: 1) a Feature Extraction Branch (FEB) to obtain feature maps with different receptive fields; 2) a Channel Enhancement Branch (CEB) to extract the boundary information of different channels; and 3) a Boundary Activation Branch (BAB) to enhance the boundary information *via* a novel activation function. In addition, in order to incorporate expert knowledge into the segmentation network, soft key point maps are generated on the choroidal boundary, and are combined with the predicted images to facilitate precise choroidal boundary segmentation. In order to validate the effectiveness and superiority of the proposed method, both qualitative and quantitative evaluations are employed on three retinal OCT datasets for choroid segmentation. The experimental results demonstrate that the proposed method yields better choroid segmentation performance than other deep learning approaches. Moreover, both 2D and 3D features are extracted for statistical analysis from normal and highly myopic subjects based on the choroid segmentation results, which is helpful in revealing the pathology of high myopia. Code is available at https://github.com/iMED-Lab/Choroid-segmentation.

## 1 Introduction

The choroid is a dense vascular layer posterior of the uvea, the middle membrane of the ocular posterior segment. It plays a critical role in thermoregulation, adjustment of retinal position, and secretion of growth factor (Nickla and Wallman, 2010). The high blood flow in the choroid makes it immune to environmental conditions with various extreme temperatures. Choroidal thickness has become one of the diagnostic indicators of many ophthalmic diseases, such as high myopia, glaucoma, age-related macular degeneration, and diabetic retinopathy (Regatieri et al., 2012; Chen et al., 2014; Wang et al., 2015; Yiu et al., 2015). Takeing high myopia as an example, the percentage of Asian young people with high myopia increaed by 6.8%–21.6% over the period 2010 to 2014 (Wong and Saw, 2016). Individuals with high myopia are highly susceptible to developing pathological myopia, which is one of the leading causes of low vision and blindness (Oduntan, 2005; Cedrone et al., 2006). Therefore, choroid segmentation and choroidal thickness analysis are crucial in determining the pathogenesis and treatment strategy of ophthalmopathy.

**FIGURE 1:**
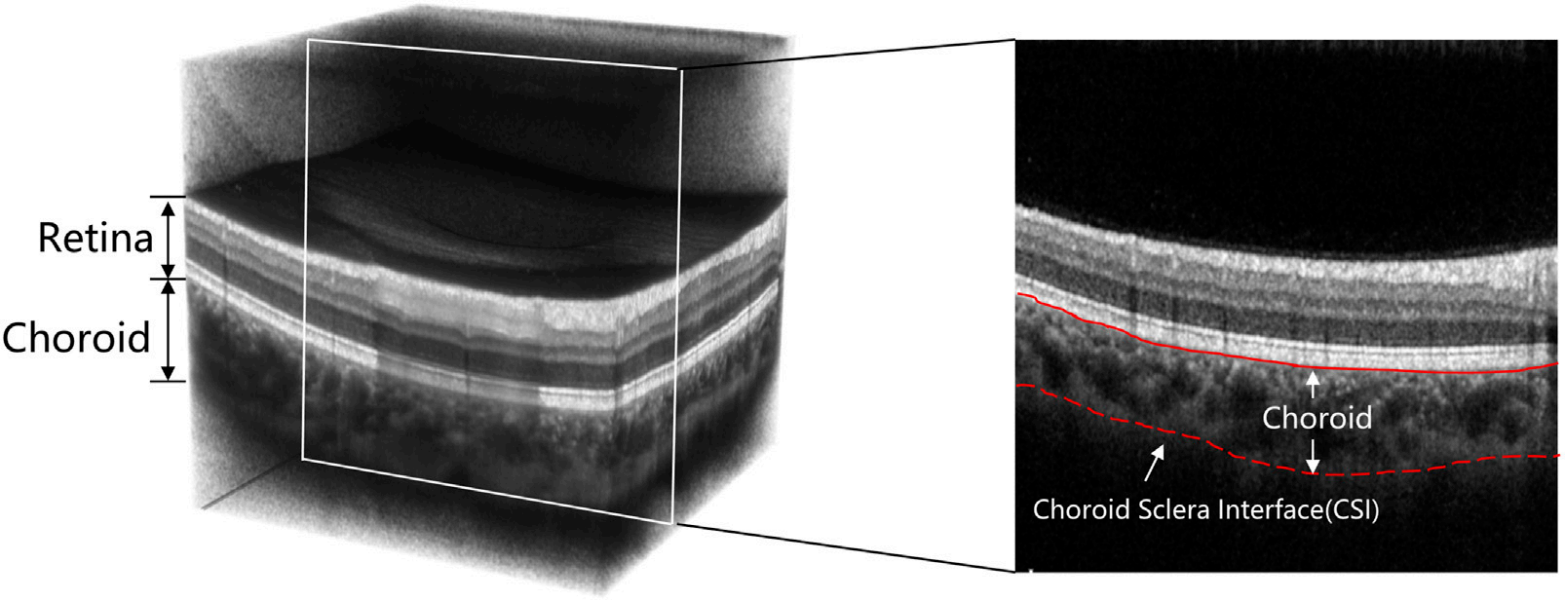
Examples of a 3D OCT volume and 2D OCT B-scan image. The choroid-sclera interface (indicated by the red dashed line) is ambiguous and difficult to extract compared to the other boundaries.

The development of Optical Coherence Tomography (OCT) (Huang et al., 1991) has made analysis of retinal and choroidal morphology convenient and accurate for clinical research and application. With the emergence of new OCT techniques such as spectral domain OCT (SD-OCT) (Yaqoob et al., 2005), enhanced depth imaging OCT (EDI-OCT) (Wong et al., 2011) and swept-source OCT (SS-OCT) (Choma et al., 2003), the choroid can be clearly visible. Because of the characteristics of non-invasive 3D imaging, these new OCT techniques have become the primary choice for clinicians to diagnose ophthalmic diseases. Figure 1 shows an OCT volume acquired from a healthy eye, which can be divided into three parts: from top to bottom (retina, choroid and sclera). In addition, the 2D B-scans can be extracted from the 3D volume for further study of the choroidal morphology.

Based on the B-scans, various methods have been proposed for choroidal layer segmentation. Previous methods were mainly based on graph theory (Zhang et al., 2012; Hu et al., 2013; Mazzaferri et al., 2017). These methods rely on manual parameter settings, and usually yield low efficiency, which limits their segmentation accuracy and makes them difficult to apply in clinical practice. With the emergence and development of deep learning, several Convolutional Neural Network (CNN) models have been applied to choroidal layer segmentation (Chen et al., 2015; Sui et al., 2017; He et al., 2021; Yan et al., 2022). The powerful feature learning capability of CNN has significantly improved choroid segmentation accuracy and efficiency over the last decade. In addition, end-to-end networks have enabled models to take original images as direct input, and output segmentation results without handcrafted operations (Mao et al., 2020; Zhang et al., 2020).

Many current studies have explored possible improvements of segmentation efficiency and model optimization, but only a few have focused on the structural characteristics of the choroidal layer. Due to the low contrast of OCT images (as shown in Figure 1), the boundary between choroid and sclera is ambiguous, which for many algorithms leads to inaccurate boundary localization. However, the issue of vagueness in imaging the Choroidal Scleral Interface (CSI) has been little investigated. Moreover, choroidal thickness, as an alternative and important biological indicator strongly associated with several ocular diseases, has been quantified in much recent work on the basis of 2D B-scans only (Regatieri et al., 2012; Kim et al., 2013; Agrawal et al., 2020). In contrast, the 3D morphological characteristics of the choroid and its thickness in different regions, such as the nasal side and the foveal region, may provide more indicative and accurate information for diagnosis of ocular diseases. But few research works have investigated choroid characteristics derived from 3D morphology.

In this paper, we focus on tackling the following two issues in choroid segmentation and morphological analysis. Firstly, since it is difficult to extract the boundary between choroid and sclera due to low contrast in OCT, existing segmentation methods usually perform ineffectively and produce poor definition of the choroidal boundary. Secondly, there is a lack of choroid-related biomarkers in highly myopic subjects, especially three-dimensional biomarkers, which are more conducive to the diagnosis and treatment of diseases.

To this end, we propose a fully automated choroid segmentation framework with boundary feature enhancement. Initially, in order to extract accurate boundary information, we design a new Boundary Enhancement Module (BEM). This consists of three parallel branches. One branch is a Feature Extraction Branch (FEB), which uses dilated convolution (Yu and Koltun, 2015) with different dilation rates to acquire relevant image features under different receptive fields, so that the boundary features are fully retained. The second branch is a Channel Enhancement Branch (CEB), which exploits and enhances the boundary characteristics of different channels through global average pooling and convolution operations. The third branch is a Boundary Activation Branch (BAB), which strengthens the boundary information from the spatial perspective *via* one-dimensional convolution and a specific activation function to further enhance boundary features. The BEM can be integrated with different encoder-decoder networks, such as the U-Net and FCN. In addition, for each B-scan, a soft point map is generated based on the extracted points on the choroidal boundary by using a boundary strengthen point selection algorithm. Based on these boundary soft point maps, we introduce the Boundary Perceptual Loss (BP-Loss) to provide feedback on the boundary enhancement effect of the output segmentation result. Finally, we extract and analyze both 2D and 3D morphological features of the choroid in the highly myopic population, based on the segmentation results.

In brief, our main contributions are listed as follows:

• We propose a novel BEM module for reinforcing information on the choroidal boundary from three perspectives including feature, channel and space, which can be integrated with major encoder-decoder architectures such as U-Net, FCN, etc.
• A boundary perceptual loss is introduced to incorporate expert knowledge into our segmentation network. This new loss provides the flexibility to learn a prior boundary information from a soft point map.
• We extract 3D edge point cloud features and reconstructed the 3D structure of the choroid based on the 2D segmentation results of all B-scans. In addition, we statistically analyze choroidal thickness and 3D characteristics in different subfields to further determine the correlation between choroidal morphological changes and high myopia.

## 2 Related work

### 2.1 Choroid segmentation

Existing methods for choroid segmentation in OCT are mainly divisible into two categories: traditional methods, and machine learning methods. Zhang et al. (Zhang et al., 2012) first attempted to extract the choroidal layer in 3D SD-OCT by adapting a graph-based method, which produced a relatively accurate choroidal surface. However, this method was tested on normal subjects only, and it is difficult to achieve the expected segmentation performance for some patients, especially those with large changes of choroidal morphology. In order to overcome this limit, Hu et al. (Hu et al., 2013) improved the graph-based multi-layer segmentation method by applying various smoothness and interaction constraints to different choroidal layer structures. This method has been validated on OCT images collected from both healthy subjects and non-neovascular AMD subjects, revealing great similarities with manual segmentation.

With the emergence of Enhanced Depth Imaging OCT (EDI-OCT) technology, its high-resolution imaging made the choroidal layer structure more clearly displayed in OCT B-scans, which is more conducive to choroid segmentation. Tian et al. (Tian et al., 2013) adopted Dijkstra’s algorithm to seek the shortest path, and the choroidal surface was quickly and accurately detected. Similarly, Danesh et al. (Danesh et al., 2014) proposed a segmentation method based on the Gaussian mixture model, to obtain the choroidal structure in EDI-OCT images. However, this still requires handcrafted features, and is sensitive to noise artifacts existing in EDI-OCT images. In addition, Chen et al. (Chen et al., 2015) introduced a new pipeline composed of a progressive intensity distance image generation algorithm and graph search method for the problem of noise and boundary ambiguity. Wang et al. (Wang et al., 2017) used a Markov Random Field (MRF) method to connect adjacent pixels, and a level set method to regularize the distance of uneven textures.

Since graph search technology is greatly affected by manual parameter settings, deep learning-based methods have been developed to obtain the choroidal structure. Sui et al. (2017) proposed a convolutional neural network (CNN)-based method that learns a graph-edge weight directly from raw OCT pixels. The network structure can be divided into two parts: one detects the CSI boundary, and the other detects the BM boundary. This method has revealed good adaptability to 3D EDI-OCT images collected from both healthy subjects and patients with macular edema. He et al. (2021) combined CNN and a *l*_2_-*l*_*q*_ (0 < *q* < 1) fitter to segment the outer choroidal surface, in which the CNN is used to generate predicted values, and the *l*_2_-*l*_*q*_ fitter is employed to maintain the stability of the fitting function. The OCT image is partitioned into small patches to form the input of the CNN, and post-processing is required to discard irrelevant information, which leads to relative inefficiency compared to end-to-end architectures. Similarly, Masood et al. (2019) used deep learning methods to establish a new segmentation structure to obtain the outer choroidal surface. Before being fed into the CNN, the OCT image needs to be divided into patches for data sampling and conversion. This method reduces the average segmentation error, but it is still not as efficient as end-to-end architectures.

As a result, several end-to-end deep learning approaches have been proposed more recently. Zhang et al. (2020) proposed an end-to-end method, consisting of a global multi-layer segmentation block, a choroidal layer segmentation block, and a regularization block. This first segments all the inner retinal layers, and then utilizes global information to detect the choroidal layer. For 3D OCT images collected from healthy subjects, the thickness difference obtained by this method (4.30 ± 0.02 pixels) is more accurate than those obtained by other state-of-the-art methods. Chai et al. (2020) proposed a method that can effectively segment the choroidal boundary by minimizing the differences between different regions, and takes into account the differences between different OCT acquisition equipment. It feeds OCT images from different domains into a U-Net-based network, and uses both adversarial and perceptual loss for domain adaptation.

### 2.2 Choroidal thickness analysis

Examining the choroidal layer as extracted from OCT images, ophthalmologists can analyze choroidal variations from different perspectives. In particular, choroidal thickness is of great interest, as it often indicates the presence or even severity of some ophthalmic diseases. Yiu et al. (2015) extracted choroidal thickness from EDI-OCT images collected from subjects with Age-related Macular Degeneration (AMD). Employing on a semi-automatic segmentation method, they analyzed the similarities and differences in choroidal thickness between normal individuals and patients with AMD. Wang et al. (2015) compared choroidal thickness between patients with high myopia and healthy people. By analyzing the experimental results, they found that choroidal thickness in healthy individuals is significantly thicker than that of individuals with high myopia. Regatieri et al. (2012) examined choroidal thickness in diabetic patients and found that the change of choroidal thickness was related to the severity of diabetes. More recently, several studies have shown that choroidal thickness as revealed by retinal OCT images is associated with certain neurodegenerative diseases. Moschos and Chatziralli (2018) extracted the retinal thickness and choroidal thickness of patients with Parkinson’s Disease (PD) from spectral domain OCT, and compared the results with those from healthy individuals. The differences between people with, and without PD were statistically significant. Similarly, Satue et al. (2018) used swept-source OCT to measure retinal and choroidal thickness of patients with PD. They found that the retina of patients with PD became thinner, while choroidal thickness might increase. Similar to our work, Chen et al. (2022) segmented the choroidal layer of highly myopic patients and non highly myopic people and compared the thickness, while they lacked the analysis of three-dimensional features, and the segmentation performance needs to be improved.

To this end, the automatic and accurate quantification of choroidal thickness is potentially crucial to diagnosis of these diseases. However, most quantification approaches of choroidal thickness can only provide two-dimensional measurements at a fixed location, which limits the practicability. Therefore, we proposed to use 3D edge point cloud features to produce a three-dimensional reconstruction of the choroidal layer.

### 2.3 Boundary segmentation

Boundary segmentation in images remains a research hotspot, not only in the fields of medical image analysis but also in many fields of other computer vision such as remote sensing. The mainstream boundary segmentation approaches may be divided into two categories: filtering-based methods, and learning-based methods. Wang et al. (2018) introduced an interactive geodesic method based on CNN into medical image segmentation: a mannual correction of boundary information is required to improve the accuracy of boundary segmentation. Lee et al. (2020) proposed a novel network with boundary preserving blocks to retain the boundary information *via* learning proper weights of boundary features. Wei et al. (2021) proposed a concentric loop CNN with a boundary detector and a refinement block to improve the effect of boundary segmentation in remote sensing images. Dang and Lee (2021) improved the effect of boundary segmentation in document images by sharing the weights of boundary features and global features, and using adversarial loss to strengthen the learning of boundary information. Wang et al. (2021) combined a transformer with CNN to enhance the segmentation of skin lesions, and used an attention mechanism to boost the performance of boundary segmentation. Recently, Yu et al. (2022) proposed FBCU-Net, which uses boundary semantic features to segment medical images, but it is mainly used for region segmentation and the performance of layer structure segmentation still needs to be improved.

## 3 Methods

**FIGURE 2:**
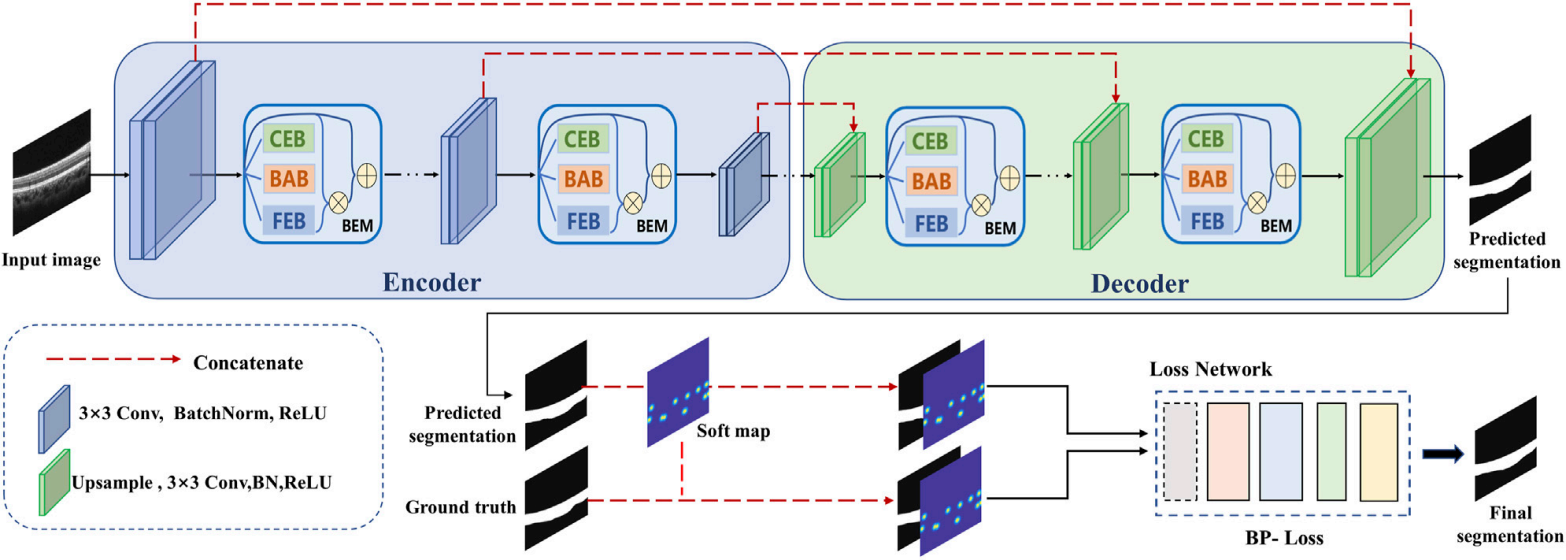
An overview of the proposed boundary enhancement framework for choroid segmentation. A novel BEM is incorporated into each encoder/decoder layer of the proposed framework. In addition, a pre-trained VGG network is utilized to calculate the specific boundary perceptual loss to improve the choroidal boundary segmentation with the guidance of the soft point map generated from ground truth.

In this paper, we propose a novel encoder-decoder network with boundary feature enhancement for choroid segmentation. The proposed choroid segmentation framework is presented in Figure 2. The proposed framework adopts U-Net (Ronneberger et al., 2015) as the baseline encoder-decoder network, and incorporates a novel module, termed a BEM into each encoder/decoder layer. The BEM consists of three parallel branches: FEB, CEB and BAB. In addition, pre-trained VGG-19 is utilized to calculate the specific boundary perceptual loss, which guides the segmentation framework in reducing the gap at boundary feature level between the predicted segmentation map and ground truth.

In order to train the proposed framework, a soft point map is constructed for each B-scan as another ground truth for extra supervision. Boundary enhancement points are first extracted using the boundary enhancement point selection algorithm. To allow tolerance of the key points’ position in the training phase, we generate Gaussian distributed disks based on all extracted points for each B-scan to construct the corresponding soft point map. The details are illustrated in the following subsections.

### 3.1 Soft point map construction

**FIGURE 3:**
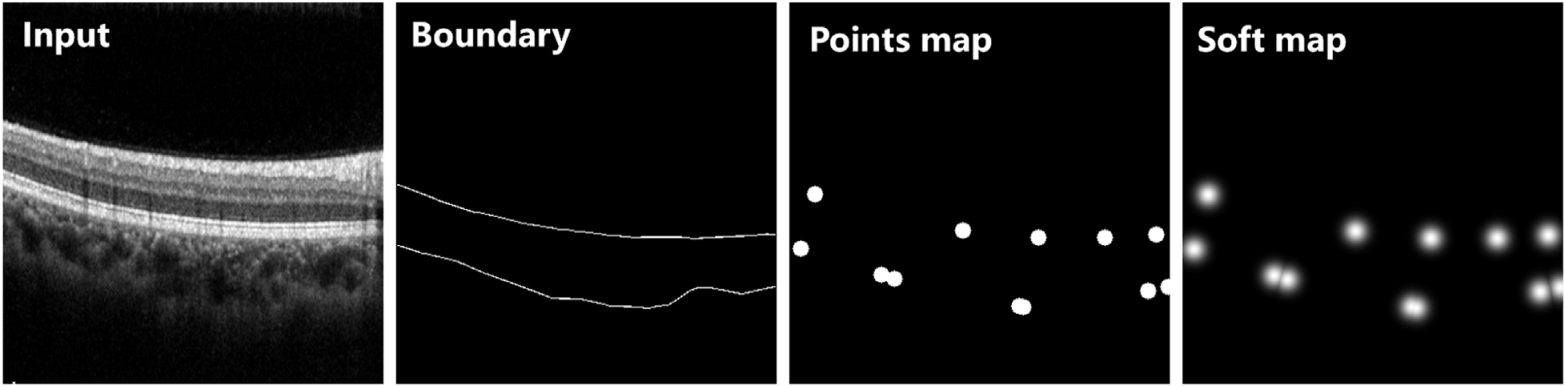
Two different types of point maps extracted from the same OCT B-scan. The original points map was generated using binary disk as (Lee et al., 2020), while the soft map was generated based on a two-dimensional Gauss function. All points were extracted from the same boundary of ground truth.

Inspired by Lee et al. (2020), we employed the boundary enhancement point selection algorithm to select several key points, then generated a point map for each B-scan as another ground truth for training. By contrast with the binary disks generated on the selected points in (Lee et al., 2020), we adopted a two-dimensional Gauss function to generate a point map with soft boundaries for more effective guidance with boundary information to segmentation. This modification is mainly based on the following considerations: a binary disk allocates undifferentiated attention to all pixels in the neighborhood of the corresponding selected point, which creates vulnerability to deviation of boundary localization. The soft point map can be expressed as follows:

$$S_{i,j}=\underset{k\in \left\{1,\ldots ,K\right\}}{\max}\exp\left(-\frac{{\left(i-x_{k}\right)}^{2}+{\left(j-y_{k}\right)}^{2}}{2\sigma^{2}}\right)$$ (1)

where (*i*, *j*) represent the coordinates of one pixel of the generated soft point map matrix *S*; (*x*_*k*_, *y*_*k*_) represent the coordinates of *k*th selected key point (total K selected key points); *σ* represents the standard deviation of the Gauss function. The differences between the original point map and the proposed soft point map are illustrated in Figure 3.

### 3.2 Boundary enhancement module

**FIGURE 4:**
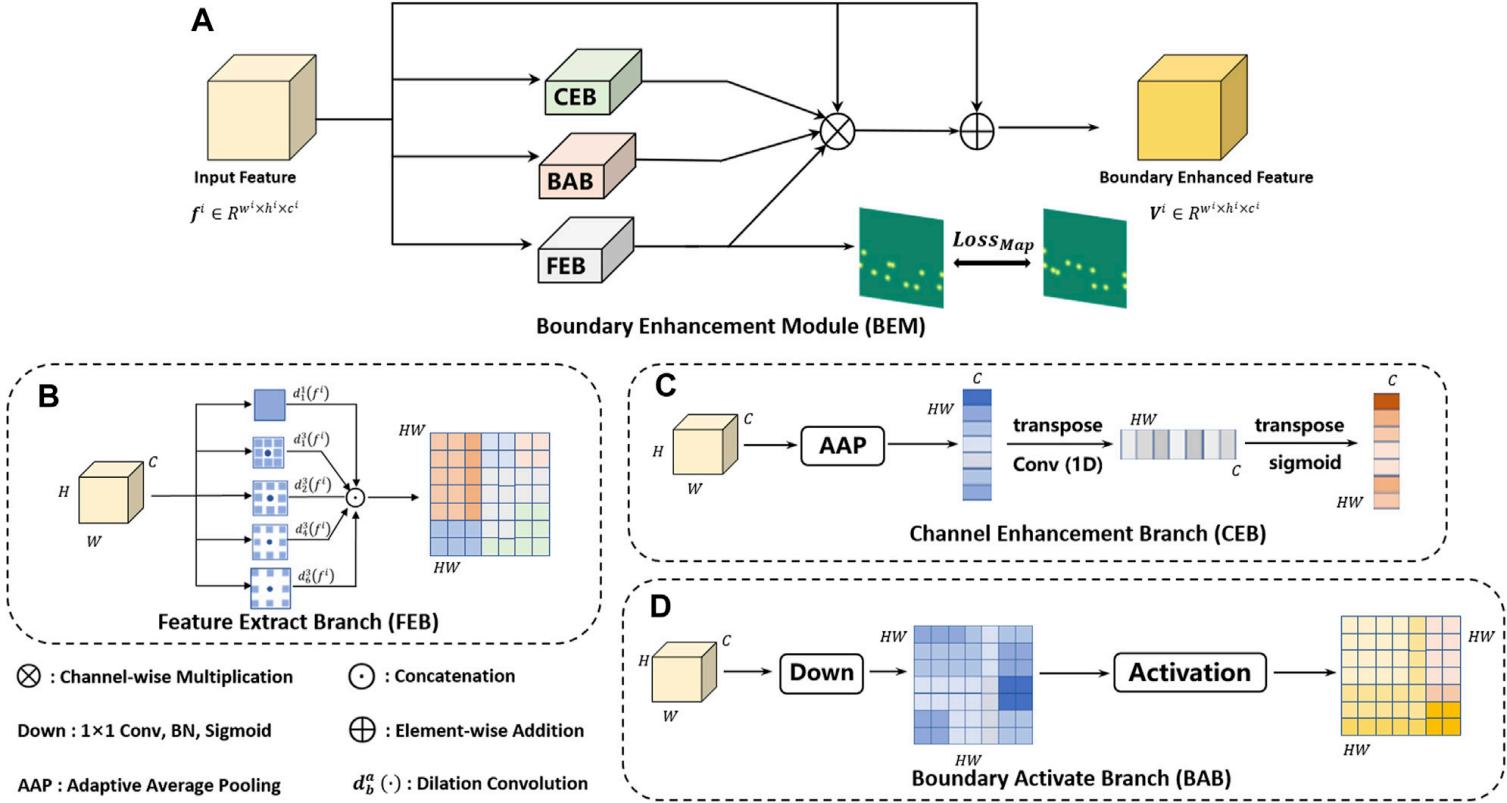
The architecture of the proposed BEM **(A)**. The BEM consists of three parallel branches including FEB **(B)**, CEB **(C)** and BAB **(D)**, which achieve enhancement of boundary information from the feature, channel and spatial perspective, respectively.

The proposed choroid segmentation architecture incorporates our novel BEMs into its encoder/decoder layers, as shown in Figure 2. Figure 4 presents the architecture of the BEM, which consists of three branches including FEB, CEB and BAB. The BEM can be embedded in various layers in the segmentation network. The BEM embedded in the *i*th layer takes the feature maps $f^{i}\in R^{w^{i}\times h^{i}\times c^{i}}$ as input, where *w* ^*i*^, *h* ^*i*^, and *c* ^*i*^ respectively represent the width, height and channels of the feature maps at the *i*th layer. The FEB produces the boundary-enhanced point map $M^{i}\in R^{w^{i}\times h^{i}\times 1}$ . The CEB generates a channel-wise weighting vector $N^{i}\in R^{c^{i}}$ . The BAB outputs a single-channel activation map $Q^{i}\in R^{w^{i}\times h^{i}\times 1}$ . Then, the final output feature maps $v^{i}\in R^{w^{i}\times h^{i}\times c^{i}}$ is calculated as follows:

$$v^{i}=f^{i}⊕\left(f^{i}⊗M^{i}⊗Q^{i}⊗N^{i}\right),$$ (2)

where ⊕ represents element-wise addition; ⊗ represents multiplication (pixel-wise multiplication for single-channel maps *M* ^*i*^ and *Q* ^*i*^, and channel-wise multiplication for the weighting vector *N* ^*i*^).

### 3.2.1 Feature extraction branch

The gray section in Figure 4 shows the architecture of the FEB, which is designed for extracting a boundary-enhanced point map with different receptive fields. Multiple receptive fields integrate global context information and local detailed information, which is beneficial to accurate localization of boundary key points. We adopted convolution with different dilation rates to obtain the features with different receptive fields. Finally, the features with different receptive fields are concatenated and then fed into a single 1 × 1 convolutional layer with a Sigmoid function. Let $d_{r}^{s}\left(f^{i}\right)$ be the encoded feature maps of input feature maps *f* ^*i*^ using *s* × *s* convolution with dilation rate *r*. Then the generated boundary-enhanced point map can be expressed as:

$$M^{i}=S\left(d_{1}^{1}\left[d_{1}^{1}\left(f^{i}\right)⊙d_{1}^{3}\left(f^{i}\right)⊙d_{2}^{3}\left(f^{i}\right)⊙d_{4}^{3}\left(f^{i}\right)⊙d_{6}^{3}\left(f^{i}\right)\right]\right),$$ (3)

where ⊙ and S denote a concatenation operation, and a Sigmoid function, respectively.

### 3.2.2 Channel enhancement branch

Each channel of a feature map may be regarded as a specific-class response. However, there are also some differences in the importance of different feature classes to a specific task (e.g., choroid segmentation). In order to selectively enhance features useful for choroid segmentation, a CEB is designed to calculate channel-wise weighting vectors for extracted feature maps. The detailed structure of CEB is shown in the green section of Figure 4. The branch first adopts global average pooling (*gap*) to generate channel-wise statistics of the input feature maps *f* ^*i*^, then applies one-dimensional convolution of kernel size 3 to obtain the final channel-wise weighting vectors *N* ^*i*^, which can be denoted as follows:

$$N^{i}=S\left(C1D_{3}\left(f_{\mathrm{gap}}\left(f^{i}\right)\right)\right),$$ (4)

where *f*_*gap*_ denotes global average pooling; *C*1*D*_3_ denotes the one-dimensional convolution of kernel size 3; S denotes a Sigmoid function.

### 3.2.3 Boundary activation branch

In order to further improve detection of the choroidal boundary, we introduced an extra branch called the BAB, as detailed in by the red section of Figure 4. The channel number of the feature maps is reduced to 1 *via* a 1 × 1 convolutional layer followed by a Sigmoid function: a specific activation function is then applied to the obtained single-channel feature map, which can be formulated as follows:

$$\left\{\begin{array}{cc} Q^{i}={e^{\left(x^{i}-0.5\right)}}^{2}+1-e^{-0.25}, & \\ x^{i}=S\left(C2D_{1}\left(f^{i}\right)\right), & \end{array}\right.$$ (5)

where *f* ^*i*^ represents the input feature maps; *C*2*D*_1_ represents the 1 × 1 convolutional layer; S denotes a Sigmoid function.

It is worth noting that the specific activation function was designed based on the observation that choroidal boundary pixels generally have a value around 0.5 in the obtained feature maps, while interior of choroid and background pixels have values near 1 and 0, respectively. To this end, the activation map is utilized to adjust each feature map spatially for the choroid segmentation task, by assigning higher weights for choroidal boundary pixels (close to 2—*e* ^−0.25^), and lower weights for interior of choroid and background pixels (close to 1) (Simonyan and Zisserman, 2014). In this way, the boundary information is highlighted after activation.

### 3.3 Loss function

In order to effectively train the proposed choroid segmentation network, we introduced a novel loss called boundary perceptual loss (BP-Loss), which embeds the soft point map into the segmentation network. The complete joint loss function is formed after incorporating segmentation loss.

### 3.3.1 Segmentation loss

First, we adopted Binary Cross Entropy Loss as the segmentation loss in order to reduce the difference between the ground-truth segmentation map and the predicted segmentation map, which is defined as:

$$Loss_{\mathrm{Seg}}=-\underset{i}{\sum }\left(\left(1-S_{GT}^{i}\right)\cdot \log\left(1-{\hat{S}}_{\mathrm{Pred}}^{i}\right)+S_{GT}^{i}\cdot \log\left({\hat{S}}_{\mathrm{Pred}}^{i}\right)\right),$$ (6)

where $S_{GT}^{i}$ and ${\hat{S}}_{\mathrm{Pred}}^{i}$ represent the *i*th pixel of ground truth segmentation map, and the corresponding predicted segmentation map, respectively.

### 3.3.2 Boundary perceptual loss

Unlike general semantic segmentation tasks, medical images require strong expert knowledge to achieve better segmentation performance. Therefore, we concatenated the generated soft point map with the predicted result and ground-truth to form the input of the VGG network, in order to constrain their geometrical relationship. To maintain consistency between the output of the FEB and ground truth. We adopted mean square error (MSE) loss and defined boundary point loss as:

$$Loss_{\mathrm{Map}}^{i}=\frac{1}{h^{i}\times w^{i}}\overset{h^{i}}{\underset{j=1}{\sum }}\overset{w^{i}}{\underset{k=1}{\sum }}{\left(M_{j,k}^{i}-M_{{GT}_{j,k}}^{i}\right)}^{2},$$ (7)

where *M* ^*i*^ and $M_{GT}^{i}$ respectively represent the output point map and the ground truth soft point map for the FEB in the *i*th layer, and *h* ^*i*^ and *w* ^*i*^ represent the corresponding height and width, respectively.

$$Loss_{GF}=\underset{i}{\sum }\frac{1}{N_{i}}\|\phi_{i}\left(S_{GT}⊙M_{GT}\right)-\phi_{i}\left({\hat{S}}_{\mathrm{Pred}}⊙M_{GT}\right){\|}_{1},$$ (8)

where *ϕ*_*i*_ (⋅) denotes the feature maps from the *i*th layer of the VGG-19 network pre-trained on the ImageNet; *S*_*GT*_ denotes ground truth segmentation map; *M*_*GT*_ denotes ground truth soft point map; ${\hat{S}}_{\mathrm{Pred}}$ denotes the predicted segmentation map; ⊙ denotes concatenation operation; and *N*_*i*_ denotes the element number of feature maps from the *i*th layer of VGG-19.

The Boundary Perceptual Loss is defined as:

$$Loss_{BP}=Loss_{GF}+\overset{n}{\underset{i}{\sum }}Loss_{\mathrm{Map}}^{i}$$ (9)

where *n* indicates the number of BEMs in the proposed choroid segmentation network.

Finally, the total loss function is defined as:

$$Loss_{\mathrm{Total}}=\lambda_{\mathrm{Seg}}Loss_{\mathrm{Seg}}+\lambda_{BP}Loss_{BP}$$ (10)

where *λ*_*Seg*_ and *λ*_*BP*_ are set as 0.5 and 0.5 in our task.

## 4 Experiment settings

### 4.1 Dateset

**FIGURE 5:**
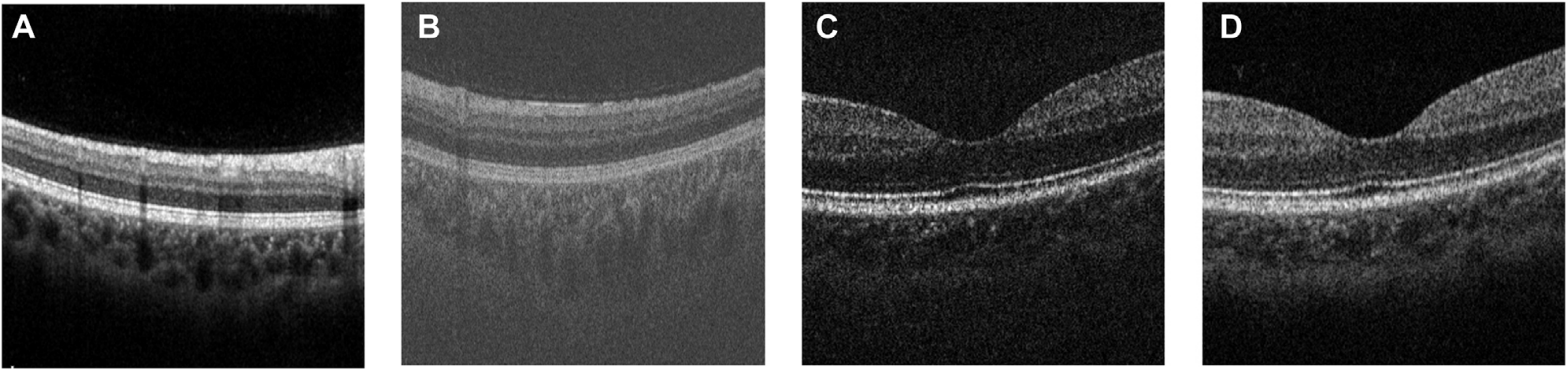
Examples of the original OCT images from **(A)** COSTA-H dataset, **(B)** COSTA-T dataset, **(C)** COSTA-B dataset (6 bit-depth), and **(D)** COSTA-B dataset (12 bit-depth).

In this work, a new **C**horoidal **O**CT image for **S**egmen**TA**tion (**COSTA**) dataset, which consists of three subsets named COSTA-H, COSTA-T and COSTA-B, was constructed for our proposed approach. These subsets were acquired from different devices or adopted different bit depths, as illustrated in Figure 5.

• **COSTA-H** consists of 10 OCT volumes from 10 healthy subjects. Each volume was captured by the Heidelberg Spectrails system, and contains 384 non-overlapping B-scans, each covering a 3 × 3 × 2 *mm* ^3^ region. Two groups of ophthalmologists were invited to independently make manual annotations of the upper and lower boundaries of the choroidal layer (BM and CSI), and their consensus were used as ground truth after discussion. In order to reduce the number of manual annotations, we asked these ophthalmologists to annotate one B-scan every six consecutive B-scans, due to the high similarity between adjacent B-scans in an OCT volume. Finally, we obtained a total of 384/6 × 10 = 640 B-scans with manually annotated choroid boundaries.

• **COSTA-T** was captured by the Topcon DRI-OCT-1 system, containing a total of 20 OCT volumes from 20 healthy human eyes. Each volume contains 256 B-scans with a resolution of 512 × 992 pixels covering a 6 × 6 × 2 *mm* ^3^ region. This dataset was also annotated by the same protocol as COSTA-H, yielding a total of 256/4 × 20 = 1280 B-scans with annotated choroid boundaries.

Both COSTA-H and COSTA-T datasets were used for training and testing, where the ratio of data volume between the training and testing sets is 3:1. To more accurately and credibly evaluate the proposed network, we adopted the 4-fold cross-validation strategy, i.e., randomly dividing all samples into 4 equal pieces and taking each piece as the validation set and others as the training set in turn. After 4 groups of tests, different validation sets are replaced each time. That is, the results of four groups of models are obtained, and the average value is taken as the final result.

• **COSTA-B** was captured by a homemade 70-Khz SD-OCT system with different bit depths, and all this data was selected from Hao et al. (Hao et al., 2020). It contains 199 annotated B-scans with a resolution of 270 × 450 pixels from one normal subject. By contrast with COSTA-H and COSTA-T, COSTA-B was only used to test the robustness of the proposed method with respect to imaging quality. For the same B-scan, we also made a comparison of segmentation results based on different bit depths. The higher bit depth represents the better image quality, which makes for easier choroid segmentation.

### 4.2 Implementation

All deep learning approaches in the experiments were implemented with PyTorch (Paszke et al., 2019) and ran on a single NVIDIA GeForce GTX 3090 GPU with 24 GB memory under an Ubuntu 16.04 system. The proposed network was trained with 400 epochs, and some hyper-parameters were set as follows: Adam optimization, with an initial learning rate of 0.0005 and batch size of 8. For other comparison methods, we adopted the same training strategy in the original paper.

### 4.3 Quantitative evaluation metrics

In order to compare the performance of the proposed method with other state-of-the-art deep learning networks, the following routine metrics for image segmentation were adopted and calculated: 

• Dice Coefficient (Dice) = $\frac{2\times TP}{2\times TP+FP+FN}$ ;

• Intersection over Union (IoU) = $\frac{TP}{TP+FP+FN}$ ;

• Accuracy (Acc) = $\frac{TP+TN}{TP+TN+FP+FN}$ ;

• Sensitivity (Sen) = $\frac{TP}{TP+FN}$ ;where TP is true positive, FP is false positive, TN is true negative, and FN is false negative.

In addition, we adopted Average Unsigned Surface Detection Error (AUSDE) (Xiang et al., 2018) based on BM and CSI:

$$AUSDE=\frac{1}{m}\overset{m}{\underset{i=1}{\sum }}|y^{\left(i\right)}-{\hat{y}}^{\left(i\right)}|$$ (11)

where *m* represents the width of the B-scan, *y* ^(*i*)^ and ${\hat{y}}^{\left(i\right)}$ represent vertical coordinates of the *i*th point in the horizontal direction of BM or CSI in the predicted segmentation map and ground truth, respectively. Based on *y* ^(*i*)^ and ${\hat{y}}^{\left(i\right)}$ of BM and CSI (respectively denoted as $y_{\text{BM}}^{\left(i\right)}$ , ${\hat{y}}_{\text{BM}}^{\left(i\right)}$ and $y_{\text{CSI}}^{\left(i\right)}$ , ${\hat{y}}_{\text{CSI}}^{\left(i\right)}$ ), average Thickness Difference (TD) can also be calculated as:

$$TD=\frac{1}{m}\overset{m}{\underset{i=1}{\sum }}\|y_{\text{CSI}}^{\left(i\right)}-y_{\text{BM}}^{\left(i\right)}|-|{\hat{y}}_{\text{CSI}}^{\left(i\right)}-{\hat{y}}_{\text{BM}}^{\left(i\right)}\|$$ (12)

## 5 Results

In this section, we performed training, validation as well as testing on COSTA-H and COSTA-T datasets, and compared them with the state-of-the-art choroid segmentation methods from both qualitative and quantitative perspectives. In addition, we applied the model trained on COSTA-H to COSTA-B to validate the robustness of the proposed method. Furthermore, we applied the proposed method to high myopia subjects. Segmentation results of all B-scans were then utilized for 3D reconstruction, which extracts 3D features for clinical correlation analysis.

### 5.1 Qualitative results

**FIGURE 6:**
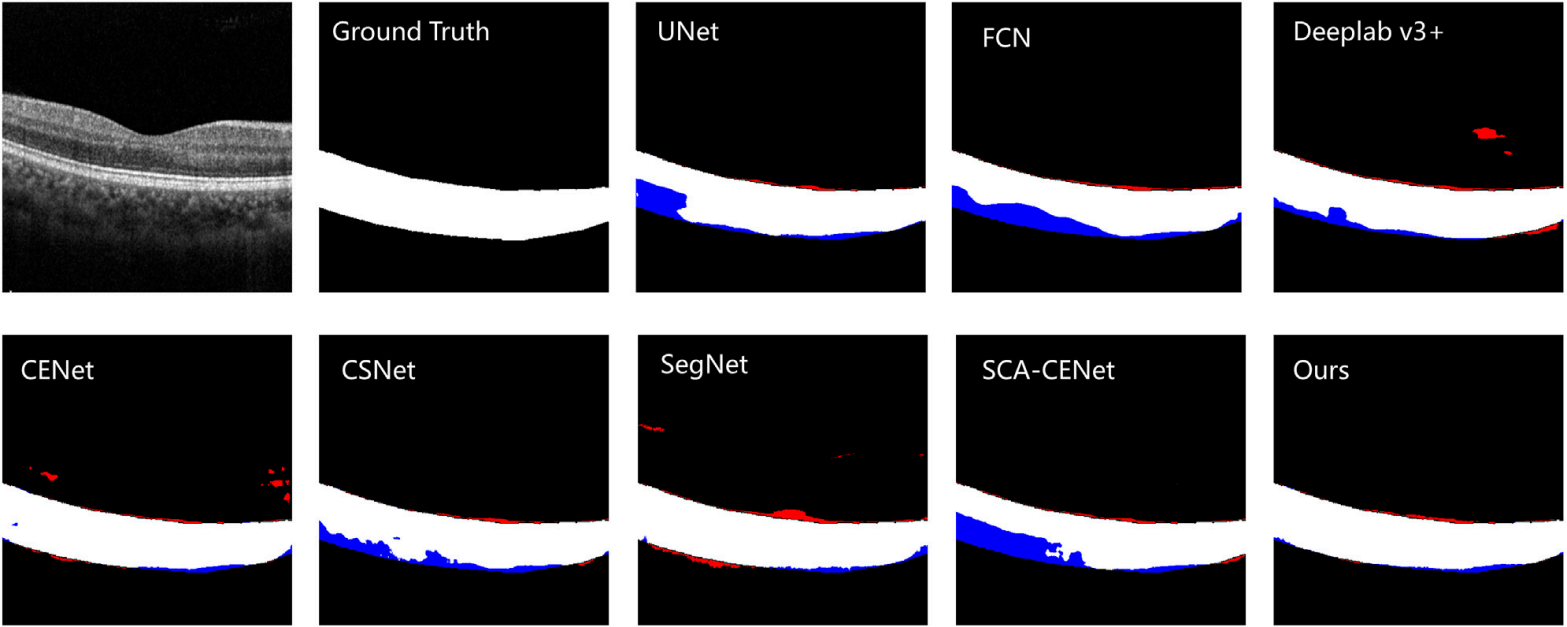
The visualization of the example result of choroid segmentation on the COSTA-H dataset. The first image is the original image, the second image is the ground truth, and the next few images are the results of different methods of segmentation: the specific methods are marked in the upper left corner of the image. White denotes a correctly segmented choroidal area, red denotes over-segmentation, and blue denotes under -segmentation.

**FIGURE 7:**
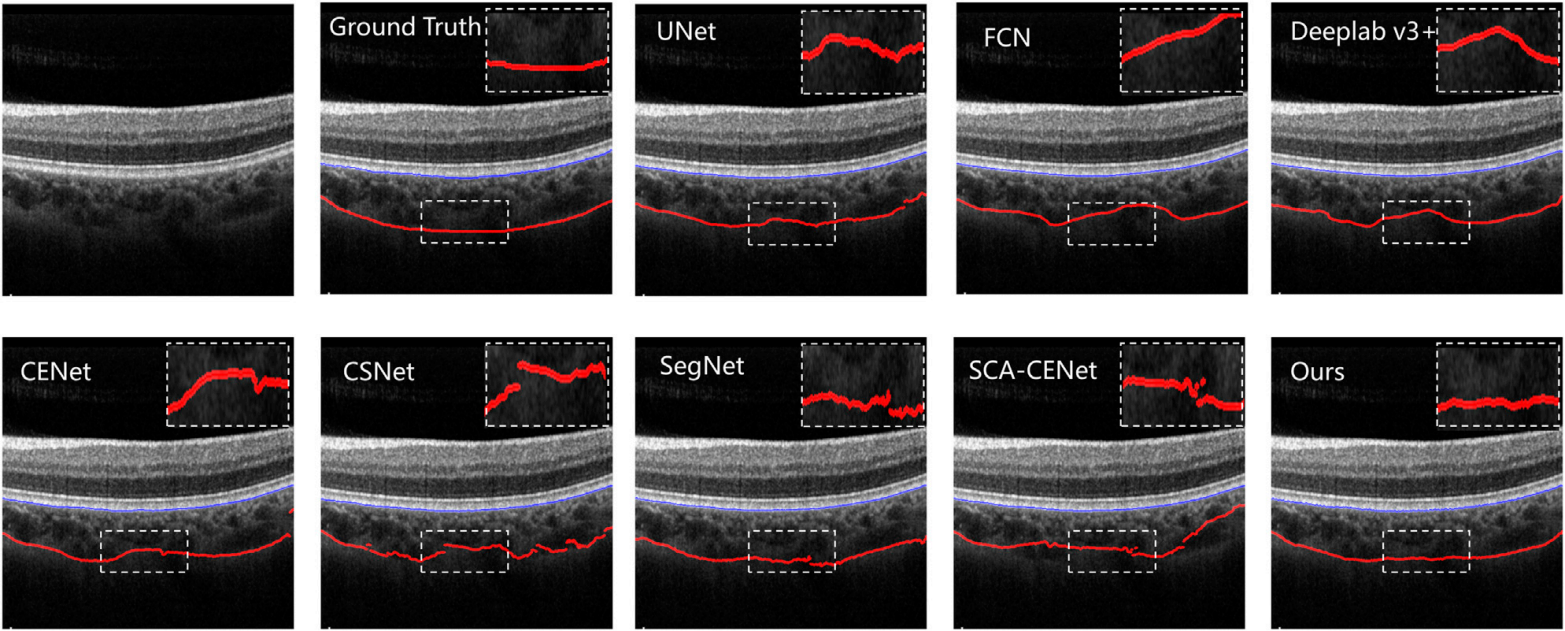
Results of different choroid segmentation methods in boundary detection. The name of the method is shown at the upper left corner of each image.

Figure 6 shows the visualization of the results of training and testing on Heidelberg, and we compare them with other popular methods that use deep learning to segment the choroid layer, including the U-Net (Ronneberger et al., 2015), FCN (Long et al., 2015), DeepLab v3+ (Chen et al., 2018), SegNet (Badrinarayanan et al., 2017), CE-Net (Gu et al., 2019), CS-Net (Mou et al., 2019), and SCA-CENet (Mao et al., 2020). In Figure 7, the BM and CSI are located by blue and red lines, respectively. In both the overall segmentation and the boundary extraction (shown by the zoomed-in part of Figure 7), the proposed BEM performed better than its counterparts, which shows that our reinforcement of boundary characteristics is useful and efficient.

**FIGURE 8:**
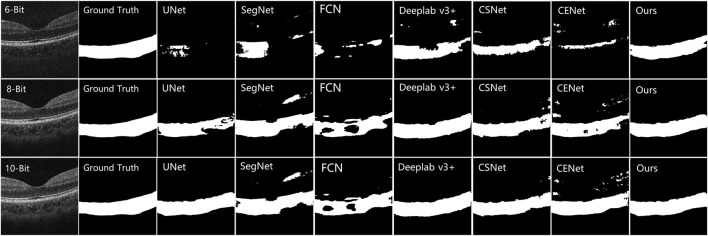
Comparison results of other choroid segmentation methods in boundary detection. The specific methods are marked in the upper left corner of the image.

The testing results on the COSTA-B dataset are presented in Figure 8, which shows three OCT B-Scans with different bit depths, including 6, 8, and 12 (the best image quality) bits. The model with the best segmentation results on the COSTA-H dataset was used to segment these images. The segmentation results show the excellent robustness of the proposed model. Common segmentation methods that focus on global information and lack detailed features have difficulty in fully segmenting all the choroid layers. In contrast, the proposed method, which benefits from its ability to enhance boundary characteristics and extract different features from different perceptual fields and channels, shows good robustness across images of differing qualities.

### 5.2 Quantitative results

In order to verify the advantages of our method from a quantitative perspective, we selected the Dice, IoU, AUSDE and TD as evaluation metrics. For the COSTA-B test, only two metrics, Dice and IoU, were selected for evaluation, as the segmentation results of many methods could not form clear boundary lines (as shown in Figure 8).

Table 1 shows the quantitative comparison of various deep learning methods applied to the COSTA-H dataset. As is shown in Table 1, our method achieved 97.06% of the Dice coefficient and 94.31% of the IoU value, with a Boundary Error of 0.9496 for AUSDE of the BM and a Detection Error of 3.0029 for the lower boundary CSI, outperforming other methods. After adding BEM and BP-Loss to UNet, the value of AUSDE of CSI is improved by 2.57 pixels and that of TD is improved by 2.68 pixels, demonstrating the value of boundary extraction.

**TABLE 1:** Quantitative segmentation results of different deep learning methods on the COSTA-H dataset.

COSTA-H							
Methods	Acc(%)	Sen(%)	Dice (%)	AUSDE (pixels)		TD (pixels)	Size (Mb)
				BM	CSI		
U-Net	99.04 ± 0.92	95.52 ± 3.87	96.10 ± 3.96	1.01 ± 1.07	5.57 ± 5.58	5.92 ± 7.01	53.7
CE-Net	99.09 ± 0.88	96.05 ± 5.12	96.30 ± 3.08	1.05 ± 0.25	3.95 ± 5.03	4.19 ± 4.89	116.2
FCN	99.00 ± 0.95	96.33 ± 5.51	96.04 ± 3.26	1.25 ± 0.46	3.93 ± 4.82	4.24 ± 4.57	134.4
SegNet	98.86 ± 0.70	96.70 ± 2.97	95.68 ± 1.84	1.33 ± 0.73	4.72 ± 3.22	4.99 ± 3.12	176.8
DeepLab v3+	98.78 ± 0.99	95.30 ± 5.40	95.23 ± 3.29	1.37 ± 0.89	5.36 ± 5.08	5.52 ± 4.94	368.1
SCA-CENet	99.14 ± 0.80	96.11 ± 4.65	96.41 ± 3.23	1.01 ± 0.30	4.11 ± 5.15	4.40 ± 5.00	116.2
CS-Net	99.00 ± 0.85	95.31 ± 4.92	96.03 ± 2.85	1.03 ± 0.50	4.32 ± 4.45	4.46 ± 4.26	**35.8**
Our method	**99.27** **±** **0.27**	**97.07** **±** **2.01**	**97.06** **±** **0.90**	**0.95** **±** **0.59**	**3.00** **±** **1.48**	**3.24** **±** **1.37**	54.1

The values in bold represent the best of all the comparative experimental results.

In order to further evaluate our proposed method, we conducted additional tests on the COSTA-T dataset and the results are shown in Table 2. With the help of BEM, our method achieves 92.87% of the Dice coefficient and 86.91% of the IoU value: after adding the BEM and BP-Loss, this improves to 2.02% and 2.39% on the Dice and IoU values, respectively.

**TABLE 2:** Quantitative segmentation results of different deep learning methods on the COSTA-T dataset.

COSTA-T							
Methods	Acc(%)	Sen(%)	Dice (%)	AUSDE (pixels)		TD (pixels)	Size (Mb)
				BM	CSI		
U-Net	97.20 ± 1.47	91.32 ± 6.25	90.85 ± 4.29	1.99 ± 0.97	11.99 ± 7.48	11.98 ± 7.45	53.7
CE-Net	97.52 ± 1.46	93.00 ± 5.85	91.77 ± 4.70	1.96 ± 0.81	10.06 ± 6.35	10.18 ± 6.49	116.2
FCN	97.19 ± 1.47	91.49 ± 6.53	90.61 ± 5.04	2.72 ± 3.56	12.17 ± 7.54	11.97 ± 8.32	134.4
SegNet	97.37 ± 1.35	92.01 ± 6.11	91.38 ± 4.04	2.12 ± 1.17	11.43 ± 6.67	11.57 ± 6.61	176.8
DeepLab v3+	96.87 ± 1.64	87.00 ± 9.05	88.95 ± 6.01	3.22 ± 4.35	13.75 ± 9.18	13.92 ± 8.71	368.1
SCA-CENet	97.62 ± 1.52	**93.30** **±** **5.93**	92.21 ± 4.78	1.94 ± 0.70	9.56 ± 6.56	9.77 ± 6.65	116.2
CS-Net	97.31 ± 1.52	90.65 ± 6.74	91.12 ± 4.58	**1.76** **±** **0.59**	11.16 ± 7.74	11.20 ± 7.76	**35.8**
Our method	**97.85** **±** **1.25**	92.47 ± 6.13	**92.87** **±** **3.70**	1.88 ± 1.07	**9.11** **±** **6.19**	**9.23** **±** **6.28**	54.1

The values in bold represent the best of all the comparative experimental results.

**FIGURE 9:**
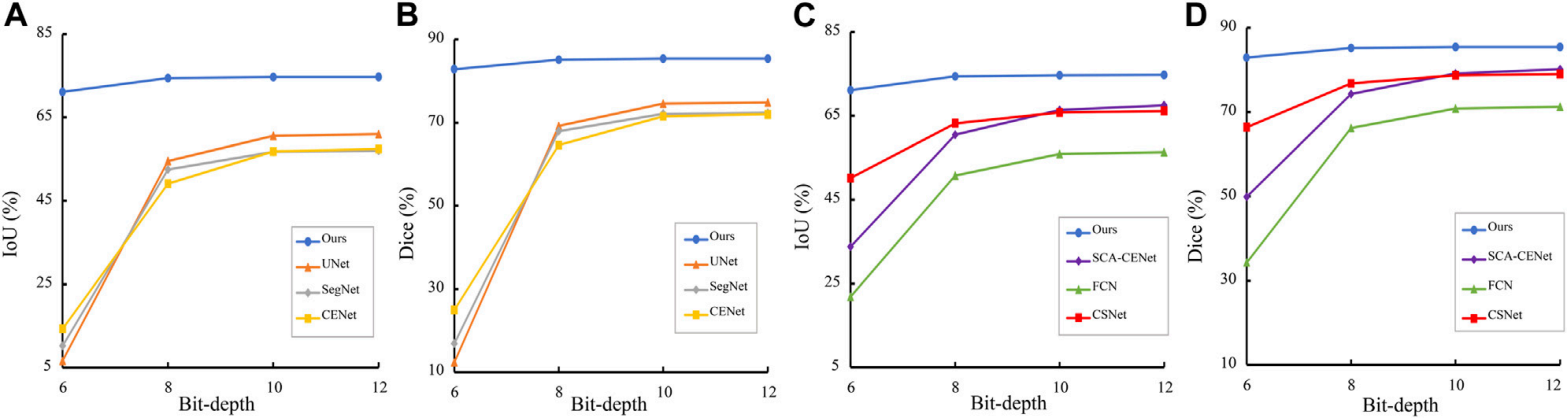
Trend of Dice and IoU results of different segmentation methods in different bit depth images. **(A)**, **(C)** are the result on the COSTA-H dataset, and **(B)**, **(D)** are the result on the COSTA-T dataset.

We also select 6, 8, 10, and 12 bit depth images in the COSTA-B dataset for evaluation on the DICE and IoU metrics. For each network, both the best trained and validated models on the COSTA-H dataset were tested. It may be seen from Figure 9, by contrast with our proposed method, the segmentation results of the other networks degrade significantly over decreased depths of the same image. Specifically, all of the tested algorithms obtain better segmentation results on the 12-bit depth map than those on the 6 and 8-bit depths. This further validates the robustness of the proposed method.

### 5.3 Ablation study

**FIGURE 10:**
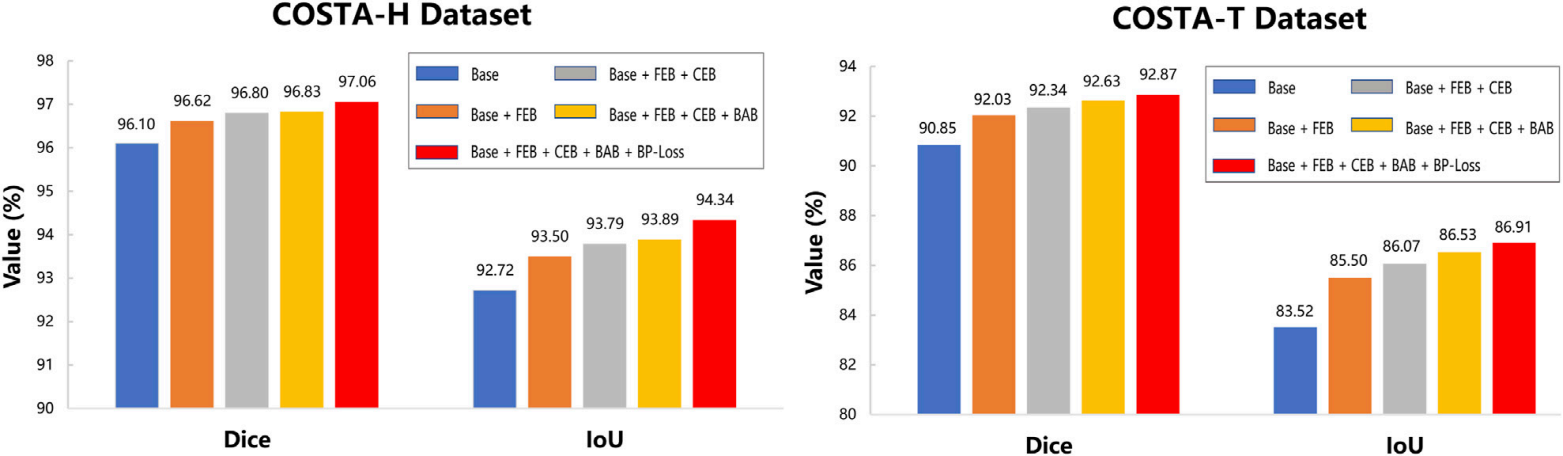
Ablation results on COSTA-H and COSTA-T datasets. The blue bars denote the quantitative results of the baseline network U-Net. The orange bars denote the segmentation results of the network with FEB. The gray bars denote the segmentation results of the network with FEB and CEB. The yellow bars denote the segmentation results of the network with FEB, CEB, and BAB. The red bars denote the segmentation results of the network with FEB, CEB, BAB, and BP-Loss.

In order to verify that each branch in the BEM and the BP-Loss are effective, we conducted ablation experiments by removing each branch separately and performing the experiments on the same dataset, and the results are shown in Figure 10. It can be seen that the dice and IoU metrics gradually increase with the addition of different branches, both in COSTA-T and COSTA-H datasets. It is obvious that the segmentation result benefits from every branch of the BEM.

After adding BEM, the IoU of the segmentation result on COSTA-T dataset reaches 86.53%, which is 3.01% higher than the baseline. With the help of BP-Loss, the segmentation result reaches 86.91%, which further improves the segmentation effect. Similarly, each module and branch plays a role on the COSTA-H dataset.

## 6 Clinic applications

High myopia is a common visual impairment worldwide. The mechanical pulling of the growing eye axis in high myopia leads to retinal and choroidal thinning, as well as to a variety of pathological changes in the fundus, which can easily evolve into pathological myopia (Read et al., 2019; Scherm et al., 2019; Singh et al., 2019). Previous studies have shown that choroidal thickness is significantly higher in highly myopic patients than that in the healthy subjects, but no correlation has been found in other features such as volume, surface area, curvature of the BM and CSI, and other 3D features. Encouraged by the good perfomance of the proposed method demonstrated in the experimentation, we applied the segmentation method to a prospective clinical study, in which the choroidal thickness of different regions are extracted and the 3D morphology of choroidal structures reconstructed using point clouds.

**FIGURE 11:**
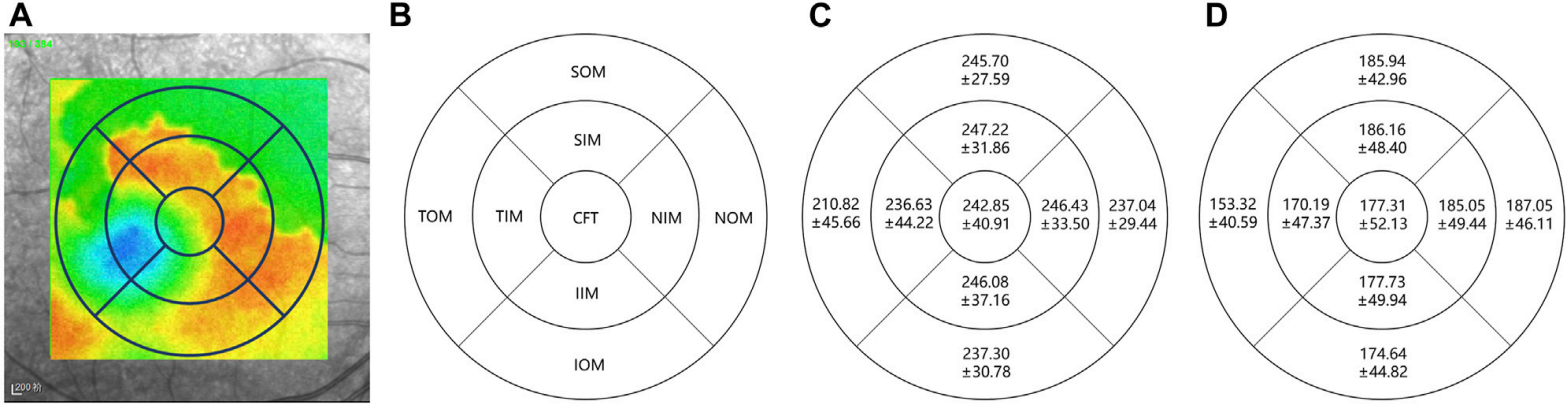
Choroidal layer thickness map in normal and highly myopic subjects using Early Treatment Diabetic Retinopathy Study (ETDRS) circles of 1 mm, 3 mm, and 6 mm. The standard ETDRS subfields dividing the macula into 9 subfields. CFT: Central foveal thickness; TIM: Temporal inner macula; NIM: Nasal inner macula; SIM: Superior inner macula; IIM: Inferior inner macula; TOM: Temporal outer macula; NOM: Nasal outer macula; SOM: Superior outer macula; IOM: Inferior outer macula. **(A)** denotes the thickness map in normal subject, **(B)** denotes the 9 subfields of macula, **(C,D)** denote the average choroidal thickness [μm] of subfields in normal subjects and highly myopic, respectively.

1) Dataset: We collected 20 volunteers aged between 20 and 30 years with high myopia. The right and left eyes of all volunteers were scanned by the Heidelberg Spectrails system device for data acquisition, and volume data were extracted within a 4.5 × 4.5 × 2 *mm* ^3^ area centered on the macular. Each volume contained 512 B-scans.

2) Result: Figure 11 shows the distribution of choroidal layer thickness in different areas across the volume, in both normal and highly myopic subjects. Table 3 shows the quantitative results of the average choroidal thickness in different subfields of the macula. The average choroidal thickness in the highly myopic subjects was significantly thinner than that in the normal subjects in all regions, with an average choroidal thickness of 230.41 ± 28.92 *μm* in the normal population and 177.31 ± 42.35 *μm* in the highly myopic subjects, while the specific thickness distribution in the other regions is shown in Table 3, with *p*-values less than 0.001 after *t*-test, which is consistent with the results reported in (Wang et al., 2015) recently.

**TABLE 3:** Average choroidal thickness and 95% CI of different Early Treatment Diabetic Retinopathy Study (ETDRS) subfields in normal and highly myopic subjects.

ETDRS subfield	Normal		High Myopia		Mean Difference (*μ*m)*	95%CI (*μ*m)		*p*-value
	Mean	SD	Mean	SD		Lower Bound	Upper Bound	
	(*μ*m)	(*μ*m)	(*μ*m)	(*μ*m)				
Center point thickness	235.93	45.54	180.94	50.72	−54.99	28.13	81.85	<0.001	
Central foveal thickness	242.85	40.91	177.31	52.13	−65.54	12.82	39.81	<0.001	
Superior inner macula	247.22	31.86	186.16	48.40	-61.06	38.97	83.16	<0.001	
Nasal inner macula	246.43	33.50	185.05	49.44	−61.38	38.55	84.22	<0.001	
Inferior inner macula	246.08	37.16	177.73	49.94	−68.36	44.32	92.39	<0.001	
Temporal inner macula	236.63	44.22	170.19	47.37	−66.44	40.79	92.08	<0.001	
Superior outer macula	245.70	27.59	185.94	48.96	−59.76	38.20	81.32	<0.001	
Nasal outer macula	237.04	29.44	187.05	46.11	−49.99	29.19	70.78	<0.001	
Inferior outer macula	237.30	30.78	174.64	44.82	−62.67	41.85	83.49	<0.001	
Temporal outer macula	210.82	45.66	153.32	40.59	−57.50	32.83	82.16	<0.001	
Global average	230.41	28.92	177.31	42.35	−53.09	33.47	72.72	<0.001	

* Normal group as reference, SD, means Standard Deviation.

Current studies on the correlation between choroidal morphology and diseases rarely involve the 3D features of the choroid. To fill this gap, we reconstructed the 3D morphology of the choroid and extracted the 3D features of choroidal volume, surface area and surface curvature using 3D point clouds. The average volume of the choroid in the central macular notch 3 × 3 × 2 *mm* ^3^ in the normal subjects was 2.211 ± 0.656 *mm* ^3^, whereas the average volume of the choroid in the same range in the highly myopic subjects was 1.304 ± 0.441 *mm* ^3^, with a *p*-value less than 0.001. This is also consistent with the relevant research results reported in (Barteselli et al., 2012).

In addition, we also calculated the surface area and the curvature of the upper and lower choroid: the results are shown in Table 4. It may be seen from Table 4 that the choroidal surface areas of highly myopic subjects and normal subjects are significantly different, while differeces of curvature between highly myopic subjects and normal subjects are not as significant.

**TABLE 4:** Results of choroidal 3D features in normal and highly myopic subjects.

	Normal		High Myopia		Mean Difference *	*p*-value
	Mean	SD	Mean	SD		
BM Curvature (*mm* ^−1^)	0.027	0.011	0.029	0.006	0.002	0.538
CSI Curvature (*mm* ^−1^)	0.091	0.024	0.115	0.141	0.024	0.474
Inner Volume (*mm* ^3^)	2.211	0.656	1.304	0.441	-0.907	<0.001
Inner Surface Area (*mm* ^2^)	1.166	0.258	0.804	0.436	-0.362	<0.001

* Normal group as reference.

## 7 Discussion and conclusion

With the emergence and popularity of deep learning methods, several choroidal layer segmentation methods have been developed during the last decade and many have been applied to choroidal segmentation tasks. However, due to the low depth and low contrast of the early OCT techniques, the applications of deep learning methods to retinal segmentation tasks have been limited. Since the continuous innovation of OCT equipment means that the choroid can now be rendered intact in B-scan, it is straightforward that the previous methods for segmenting the retinal layer should be applied to the task of choroidal layer segmentation. However, even when using the most recent technical improvements in OCT imaging, the CSI layer of the choroid is still not as clear as the boundaries of the retina. Therefore, the models developed for retinal layer segmentation tend to generate ambiguous results when applied to the choroidal boundary.

Recognizing the limitations of existing models, the goal of our work is to develop a method to automatically segment the choroidal layers, while dealing with the ambiguous boundary. We enable the segmentation network to focus on the boundary features by adding a boundary enhancement module to the major segmentation network. The module has three branches to enhance boundary features *via* different perspectives: expanding the perceptual field using dilated convolution, activating a boundary features using the boundary activation function and extracting the boundary features of different channels using channel convolution.

In order to embed expert knowledge into the proposed choroidal automatic segmentation model, we extract boundary enhancement points from the boundary of ground truth and generate a soft point map, then introduce a boundary perceptual loss, so that the boundary region information can be fed back to the segmentation network based on ground truth, following which the accurate segmentation of the choroidal layer can be performed.

In addition, in order to further validate the clinical application of this method, compared with previous studies, we investigated not only from the two-dimensional perspective of thickness, but also from a three-dimensional perspective. The differences of choroidal 3D morphological structures between highly myopic and normal subjects are compared. This paper demonstrates the effectiveness of the proposed method, which has the potential to promote understanding the pathogenesis of some eye diseases (e.g., high myopia) related to morphological changes of the choroid, so as to support early screening and intervention.

However, this work has limitations. For example, the volunteer normal subjects may have a certain degree of myopia, yet still not reach the definition of high myopia, which may affect the statistical analysis of final results. The dataset employed for validation might be extended, not only in terms of data volume but also in terms of disease types, such as glaucoma and pathological myopia. Another limitation of our method is that it is less useful for tackling multi-layer (multi-class) segmentation tasks. Since the selected boundary enhancement points have not been further classified by different layers, soft point map construction and boundary enhancement module in the proposed method might not be suitable for multi-layer segmentation in retinal OCT images. In future work, the proposed model may be improved by setting different weights to the boundary points, which would change the type and number of points adaptively. In this way, the proposed model might then be applied to both binary and multiclassification tasks.

## Data Availability

The original contributions presented in the study are included in the article/supplementary material, further inquiries can be directed to the corresponding author.
